# Influence of Mind on Disease

**Published:** 1854-11

**Authors:** 


Influence of Mind on Disease.—It would seem as if the study of certain diseases sometimes favored their real or imaginary development. Mæunnec does of phthisis, and Corvisart of disease of the heart. When the celebrated Professor Frank was preparing his lectures at Paris, on disease of the heart, his own heart became so disturbed that he was obliged to rest for awhile. Rumor says that no less than five of the professors in one of the medical colleges have unjustly suspected their hearts. Medical students, exhausted by a winter session, are apt to be special subjects of real or im·ginary irregularity of the heart. A young friend who attended the lectures last winter, on diseases of the chest, felt an unusual knocking of his heart after ascending the long college stairs, and required several examinations to satisfy him that there was no danger.—*N. Y. Med. Gaz.*

